# Immunological changes in a cohort of COVID-19 survivors: Mansoura University experience

**DOI:** 10.12688/f1000research.134565.1

**Published:** 2023-07-06

**Authors:** Tamer Elhadidy, Heba Wagih Abdelwahab, Doaa Shahin, Asem Hewidy, Eman Khashaba, Rehab Ahmad Elmorsey, Nermin Abo El Kheir, Elsayed A. Eid, Ahmed El-Mesery, Marwa O. Elmaria

**Affiliations:** 1 Chest Medicine, Faculty of Medicine, Mansoura University, Mansoura, Dakahlia Governorate, 35516, Egypt; 2 Clinical Pathology Department, Faculty of Medicine, Mansoura University, Mansoura, Dakahlia Governorate, 35516, Egypt; 3 Public Health & Community Medicine, Faculty of Medicine, Mansoura University, Mansoura, Dakahlia Governorate, 35516, Egypt; 4 Medicine and Endocrinology, Faculty of Medicine, Delta University for Science and Technology, Belkas, Dakahlia Governorate, 7730103, Egypt; 5 Tropical Medicine, Faculty of Medicine, Mansoura University, Mansoura, Dakahlia Governorate, 35516, Egypt

**Keywords:** Post COVID immunity, COVID-19, IgM, IgG, antibodies, diabetes, follow up study

## Abstract

**Background:** COVID-19 is a global pandemic that has affected millions of people all over the world since 2019. Infection with COVID-19 initiates a humoral immune response that produces antibodies against specific viral antigens, which in turn is supposed to provide immunity against reinfection for a period of time. The aim of this research was to study the kinetics of IgM and IgG antibodies against SARS-CoV-2.

**Methods:** One hundred and seventeen post-COVID-19 participants were enrolled in the study.  Qualitative assessment of IgM and IgG antibodies over six months (three visits) post recovery was conducted.

**Results:** The current study revealed a significant reduction in IgM and IgG titers between the first and second visits (p <0.001). After six months, the antibody titer had declined by 78.8% from the first visit for IgM and by 49.2% for IgG antibodies. Regarding younger age and male sex, statistically significant persistence of IgM antibodies was noticed at the six months follow up. Also, statistically significant persistent IgG immunity was found in male patients and diabetics by the end of the six months follow up.

**Conclusions:** We observed a significant waning of IgM and IgG titers over a period of six months follow up.. The persistence of positive IgM and IgG antibodies by the end of six months was variable due to differences in age, gender and presence of diabetes mellitus.

**List of abbreviations**
CEST: central European summer time
CMIA: chemiluminescent microparticle immunoassay
COVID-19: coronavirus disease 2019
IgG: immunoglobulin G
IgM: immunoglobulin M
RBD: receptor binding domain
RLU: relative light unit
SARS: severe acute respiratory syndrome

## Introduction

COVID-19 is considered the most catastrophic pandemic in the 21 ^st^ century until now. Globally, as of 3 June 2022, 5:37pm CEST, there have been 528,816,317 confirmed cases of COVID-19, including 6,294,969 deaths, reported to WHO ( WHO, 2022).

Infection with severe acute respiratory syndrome coronavirus 2 (SARS-CoV-2), the virus that causes COVID-19, initiates a humoral immune response that produces antibodies against specific viral antigens. These include anti-S protein antibodies that target the virus spike S1 protein subunit and receptor binding domain (RBD) ( CDC, 2022).

Antibodies are detected in the blood of people who have been previously infected with a virus that causes a disease; they show the body’s efforts to fight off a specific virus. Antibodies may protect people from reinfection or becoming severely ill for some time afterward ( Qu *et al*., 2020). However, antibodies wane over time and how quickly antibodies wane is different for each disease and each person.

The aim of this study was to identify the kinetics of IgM and IgG antibodies in a cohort of COVID-19 survivors over six months’ follow up and evaluate the longevity of immunity provided by COVID-19 infection.

## Methods

### Study design and setting

This longitudinal follow up study was conducted between May 2020 to April 2021 at post-COVID-19 outpatient clinics at several Mansoura University Main Hospital. Ethical approval was obtained from the Mansoura University Institutional Research Board (IRB) (approval number: RP.20.05.67). Written informed consent was taken from all participants and all study participants were assured of the confidentiality and anonymity of the data.

### Study participants

**Figure 1.**
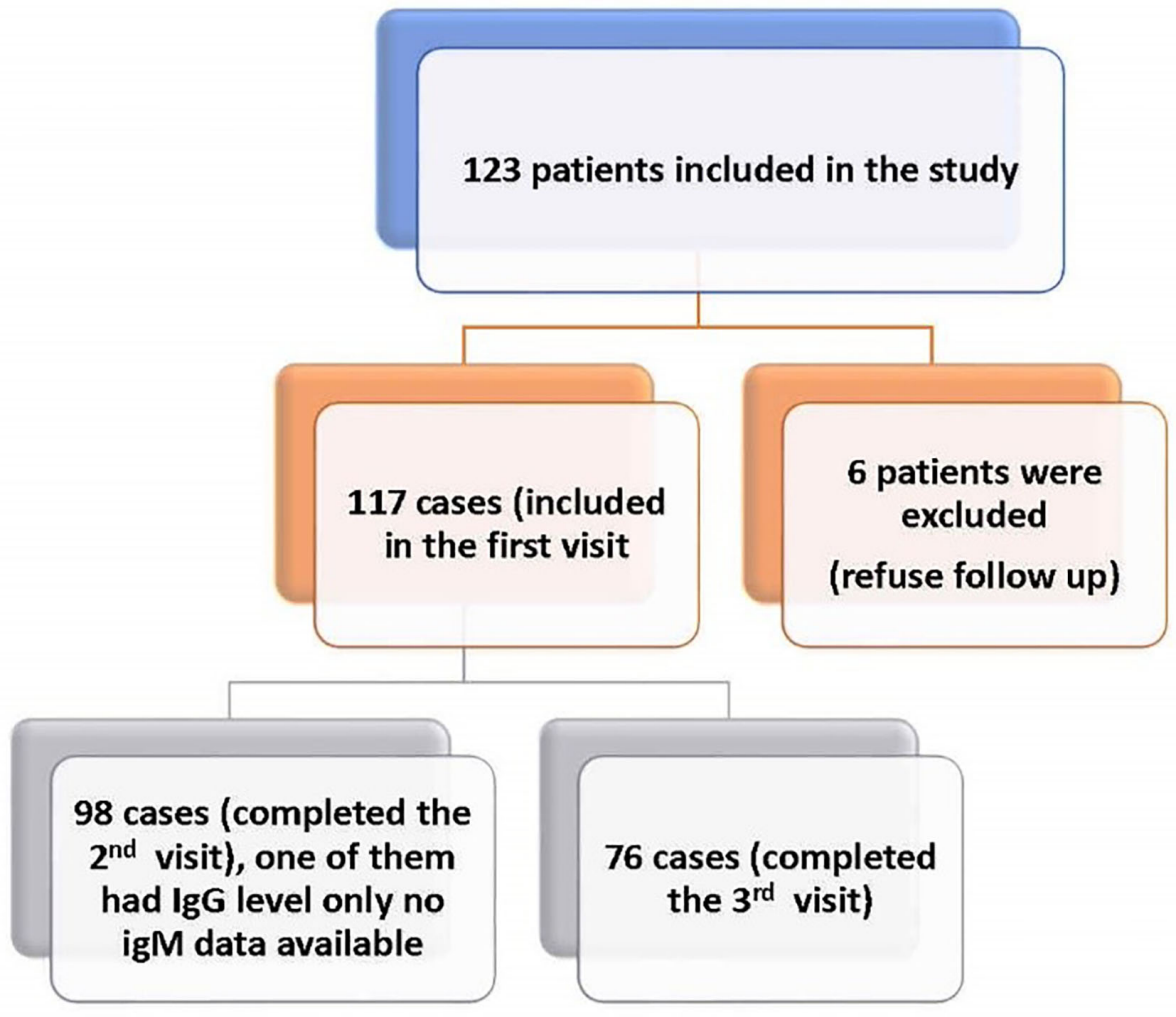
Flow chart of COVID 19 studied cases.

A convenience sample of 123 post-COVID-19 patients who attended the outpatient’s clinic were recruited. The follow up schedule was explained to them and they were free to leave the study at any time. Six participants were excluded because they refused follow up. Consequently, 117 post-COVID-19 participants were studied. Follow up was conducted at one month (first visit), three months (second visit) and six months (third visit) after recovery from COVID-19. Of 117 survivors who attended the first visit, only 98 attended the second visit and 76 completed the third visit ( Figure 1). Demographics, comorbidities, severity of previous COVID-19 and laboratory data of the participants were recorded. Severity of previous COVID-19 infection was classified according to WHO severity definitions ( WHO, 2021).

### Data collection tool

A clinical sheet was used including demographic data such as age, gender, marital status, smoking status, history relevant to clinical presentation and medical comorbidity, pulmonary function testing, electrocardiogram, necessary laboratory analysis such as complete blood picture (CBC), COVID-19 IgG and IgM antibodies titer and computed tomography (CT) chest (see Extended data ( Khashaba, 2023)).

### Chemiluminescent immunoassay

Serum samples using the Abbott SARS-CoV-2 IgG and IgM assays following the manufacturer’s instructions (SARS-CoV-2 IgG ( https://www.fda.gov/media/137383/download); SARS-CoV-2 IgM ( https://www.fda.gov/media/142940/download) (Abbott Laboratories, Abbott Park, IL, USA (reference 06R8620 and 06R8720; Abbott ARCHITECT, 2020; Abbott AdviseDx, 2020)). The assay is a chemiluminescent microparticle immunoassay for qualitative detection of IgG and IgM in human serum or plasma against the SARS-CoV-2 nucleoprotein. The amount of IgG and IgM antibodies to SARS-CoV-2 in each sample is determined by comparing its chemiluminescent relative light unit (RLU) to the calibrator RLU (index S/C). Using an index S/C threshold of 1·4 (for IgG) and 1 (for IgM) ( Bryan *et al.* 2020).

### Statistical analysis

The collected data was revised, coded, tabulated and introduced to a PC using Statistical Package for Social Science software (IBM SPSS Statistics for Windows, Version 23.0. Armonk, NY: IBM Corp). Descriptive statistics in the form of means and standard deviation (±SD) was used for normally distributed numerical data, while median and range was used for non-normally distributed numerical data. The frequency and percentage of qualitative data was recorded. The Wilcoxon signed rank test was used to compare quantitative data at different times, which was non-normally distributed. A Chi square test was used to compare qualitative data in two groups. The McNemar test was used to compare immune status at different points of time. Logistic regression analysis was used for predictors of positive immunity among the studied group using a forward Wald model. Adjusted odds ratios and their 95% CIs were calculated. A p-value less than 0.05 was considered as statistically significant.

## Results

Baseline characteristics of the cohort (117 post-COVID-19 participants) were as follow: mean age was 54 years old, about 54.7% of the participants were male, diabetes mellitus was detected in 38.5% while hypertension was detected in 34.5%. In addition, 46.2% and 47.9% of participants previously had non-severe and severe COVID-19 infection respectively (Underlying data ( Khashaba, 2023)).

Of 117 participants, only 98 were enrolled in the second visit (three months post COVID-19) of which one IgM result was not recorded. Only 76 participants completed the third visit (six months post COVID-19) ( Table 1).

**Table 1.** Post COVID-19 immunity among studied participants.

Post COVID-19 Immunity	Follow up visits				
	One month	Three months	Six months	P-value	
	Median (min–max)	Median (min–max)	Median (min–max)	*p1*	*p2*
**IgM titer of positive participants**	10.9 (1.01–69.3)	3.4 (1.02–52.8)	2.3 (1.05–19.4)	**<0.001**	**<0.001**
**IgG titer of positive participants**	6.7 (1.5–30.4)	4.3 (1.6–8.2)	3.4 (1.6–27.7)	**<0.001**	**<0.001**
**IgM no. (%)**	**n=117**	**n=97**	**n=76**		
**Positive**	103 (88.0)	69 (71.1)	41 (53.9)	**<0.001**	**0.001**
**Negative**	14 (12.0)	28 (28.9)	35 (46.1)		
**IgG no. (%)**	**n=117**	**n=98**	**n=76**		
**Positive**	109 (93.2)	78 (79.6)	46 (60.5)	**<0.001**	**<0.001**
**Negative**	8 (6.8)	20 (20.4)	30 (39.5)		

Wilcoxon signed rank test, McNemar Test.

**p1:** statistical significance between one month and three months' post-COVID-19, **p2:** statistical significance between one month and six months’ post-COVID-19.

Regarding the COVID-19 IgM antibody titer, there was significant reduction in IgM titer between the first and second visits (percentage change 68.8%; p<0.001). Moreover, a reduction in the IgM titer was observed between the second and third visit (percentage change 32.3%). After 6 months follow up (third visit) COVID-19 IgM titer declined by 78.8% from the first visit (Underlying data ( Khashaba, 2023)).

A total of 88% (103 out of 117) of participants had a positive COVID-19 IgM in the first visit and after six months follow up 53.9% (41 out of 76) participants had a positive COVID-19 IgM and the difference was statistically significant (p<0.05) ( Table 1).

For the COVID-19 IgG antibody titer, there was a significant reduction in IgG titer between the first and second visits (p<0.001). Also, a significant reduction in IgG titer was observed between the first and third visit. After six months follow up, at the third visit, the COVID-19 IgG titer had declined by 49.2% from the first visit ( Table 1).

A total of 93.2% (109 out of 117) participants had positive COVID-19 IgG at the first visit and after six months follow up 60.5% (46 out of 76) of participants had positive COVID-19 IgG ( Table 1).

The existence of a positive IgM test at the third visit was significantly associated with the age of the studied participants (mean age was 52 in participants with a positive test compared to 57.4 in participants with a negative test (p=0.04). Also, a positive test was more frequent among males than females (p=0.04). Moreover, positivity was significant among healthcare workers compared to non-healthcare workers (p=0.04). Although a non-significant association was detected between a positive IgM test and diabetes mellitus, a positive IgM test was frequent among non-diabetic participants (60.9% versus 43.3%). In addition, a positive IgM test was frequent among non-smokers and participants who had previously had severe and critical COVID-19 despite no statistical significance ( Table 2).

**Table 2.** Association between patients’ characteristics and IgM level six months’ post COVID-19.

	Positive test (IgM) n=41	Negative test (IgM) n=35	P-value	Unadjusted OR (95% CI)
**Mean age (sd)**	52.0 (12.0)	57.4 (10.2)	0.04*	---
**Sex**				
Male	27 (64.3)	15 (35.7)	0.04*	2.5 (1.0–6.5)
Female (r)	14 (41.2)	20 (58.8)		1
**Smoking**				
None (r)	36 (55.4)	29 (44.6)	0.7	1
Smoker/Ex-smoker	5 (45.5)	6 (54.5)		0.6 (0.2–2.4)
**Occupation**				
Health care workers	9 (81.8)	2 (18.2)	0.04*	4.6 (0.9–23.2)
Non health care workers (r)	32 (49.2)	33 (50.8)		1
**Diabetes mellitus**				
No (r)	28 (60.9)	18 (39.1)	0.1	1
Yes	13 (43.3)	17 (56.7)		0.5 (1.0–1.2)
**Hypertension**				
No (r)	28 (59.6)	19 (40.4)	0.2	1
Yes	13 (44.8)	16 (55.2)		0.5 (0.2–1.4)
**Severity**				
Not severe (r)	15 (48.4)	16 (51.6)	0.4	1
Severe/critical	26 (57.8)	19 (42.2)		1.4 (0.5–3.6)

Chi square test, row percent is considered.

On studying persistent IgG positivity at the third visit, it was slightly associated with older age of studied participants (mean age was 56.7 in participants with a positive test compared to 51.4 in participants with a negative test (p=0.05)). Also, a positive test was more frequent among males than females (p=0.008). A significant association was detected between a positive IgG test and diabetes mellitus as it was more frequent among diabetic than non-diabetic participants (83.3% versus 45.7%). Finally, a positive IgG test was more frequent among non-smokers, and participants previously having had severe and critical COVID-19 despite the absence of statistical significance ( Table 3).

**Table 3.** Association between patients’ characteristics and IgG level six months post COVID-19 (n=76).

	Positive test (IgG) n=46	Negative test (IgG) n=30	P value	Unadjusted OR (95% CI)
**Mean age (sd)**	56.7 (11.0)	51.4 (12.03)	0.05	-------
**Sex (%)**				
Male	31 (73.8)	11 (26.2)	0.008*	3.5 (1.4–9.4)
Female (r)	15 (44.1)	19 (55.9)		1
**Smoking no. (%)**				
None (r)	40 (61.5)	25 (38.5)	0.7	1
Smoker/Ex-smoker	6 (54.5)	5 (45.5)		0.7 (0.2-2.7)
**Occupation no. (%)**				
Health care workers	6 (54.5)	5 (45.5)	0.6	0.7 (0.2-2.7)
Non-Health care workers (r)	40 (61.5)	25 (38.5)		1
**Diabetes mellitus no. (%)**				
No (r)	21(45.7)	25 (54.3)	0.002*	1
Yes	25 (83.3)	5 (16.7)		5.9 (1.9–18.3)
**Hypertension no. (%)**				
No (r)	27 (57.4)	20 (42.6)	0.5	1
Yes	19 (65.5)	10 (34.5)		1.4 (0.5–3.6)
**Severity no. (%)**				
Not severe (r)	16 (51.6)	15 (48.4)	0.2	1
Severe/critical	30 (66.7)	15 (33.3)		1.8 (0.7–4.7)

Row percent is considered, reference group (r).

Based on logistic regression analysis, age, sex and occupation are significant predictors for positive IgM immunity; older age is associated with a decreased chance of positive IgM [OR (95%CI) 0.9 (0.8–0.9)]. However, males and health care workers had an increased chance of a positive IgM [OR (95%CI) 5.06 (1.6–16.2), 6.2 (1.0–35.5) respectively]. Regarding IgG immunity, males and diabetic participants had an increased chance of a positive IgG [OR (95%CI) [4.2 (1.4–12.3), 7.3 (2.2–24.3) respectively] ( Table 4).

**Table 4.** Predictors of COVID-19 immunity (logistic regression analysis).

Predictors	IgM at six months			IgG at six months		
	β	p-value	Adjusted OR (95% CI)	β	p-value	Adjusted OR (95% CI)
**Age** (continuous)	-0.06	0.01*	0.9 (0.8–0.9)	-	-	-
**Sex**						
Male	1.6	0.006*	5.06 (1.6–16.2)	1.42	0.01*	4.2 (1.4–12.3)
Female (r)						
**Occupation**						
Health care workers	1.8	0.04*	6.2 (1.0–35.5)	-	-	-
Non-Health care workers (r)						
**Diabetes mellitus**						
No (r)	-	-	-	1.98	0.001*	7.3 (2.2–24.3)
Yes						
**Constant–**	2.6			-1.01		
**Percent correctly predicted**	64.0			72.1		
**Model χ ^2^**	16.8; 0.001			19.3; <0.001		

**Figure 2.**
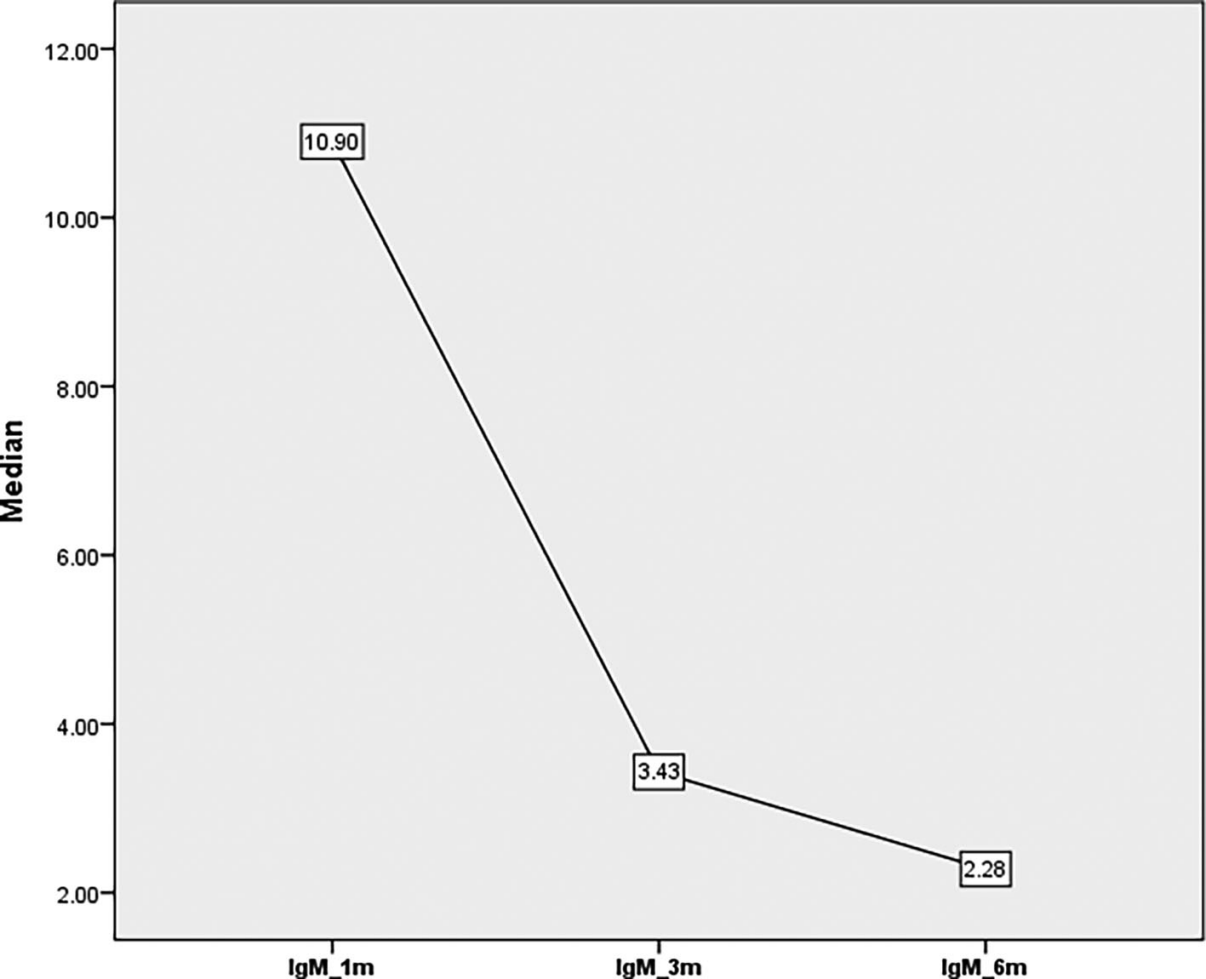
IgM titer from one month to 6 months’ post COVID-19.

**Figure 3.**
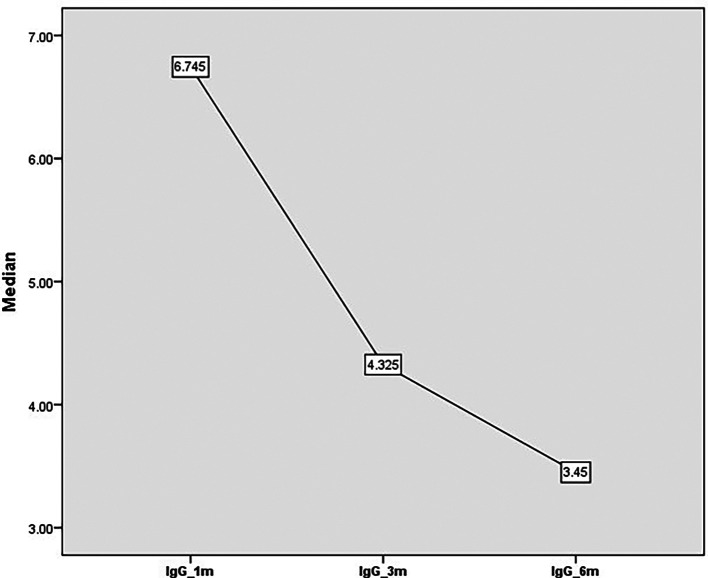
IgG titer from one month to 6 months’ post COVID-19.

There was an overall reduction in the level of IgM from 10.9 units at one month to 3.4 units after three months and 2.3 units at six months ( Figure 2). There was an overall reduction in the level of IgG from 6.7 units at one month to 4.3 units after three months and 3.4 units at six months ( Figure 3).

## Discussion

Understanding the persistence of neutralising antibodies in COVID-19 survivors is essential for developing effective vaccination programmes and COVID-19 pandemic control measures. Here, we looked into COVID-19 survivors’ natural neutralising antibody survival rates ( Greaney *et al*., 2021; Sette and Crotty, 2021). We studied the changes of IgM and IgG to the spike protein of SARS-CoV-2 in 117 post COVID-19 participants. There was significant reduction in IgM and IgG antibody titer over time. By the end of six months follow up, IgM and IgG titer declined by 70.5% and 46.8% from the first visit respectively. Forty-one out of 76 participants remained IgM positive by the end of the sixth month. When relating this positivity with different factors, there was significant relation to mean age of 52 years old and male sex. Although not statistically significant, there was persistently more positive IgM in non-smokers, non-diabetics, non-hypertensive and in more severe and critical cases. Forty-six out of 76 cases remained IgG positive by the end of the sixth month. Positive IgG immunity is found more frequently and statistically significant in older participants, male sex and diabetics. Although not statistically significant, there was persistently more positive IgG in non-smokers, hypertensive, and in more severe and critical cases.

The persistence of IgG antibodies in diabetics compared to non-diabetic participants may be explained by more severe disease in diabetic patients. This is supported by several studies which stated that ICU patients showed a faster and increased neutralising antibody response compared to non-ICU patients ( Lau *et al*., 2021; Liu *et al*., 2020; Qu *et al*., 2020; Williamson *et al*., 2020). The association between serum IgA, IgG, and IgM levels and glycated haemoglobin, an indicator of long-term diabetes control, fructosamine, and 111 healthy non-diabetic people was examined in 110 diabetic patients. While the concentration of IgM was considerably lower (by 46.7%, p<0.001) in diabetic patients compared to non-diabetic participants, there were significant increases in serum IgA (by 82.7%, p 0.001) and IgG (by 35.2%, p 0.001) concentrations ( Ardawi *et al*., 1994).

The anti-SARS-CoV-2 antibody titers were statistically higher in older compared to younger participants ( Lau *et al*., 2021; Ozgocer *et al*., 2022) and in men compared to women ( Jin *et al*., 2020; Klein *et al*., 2020; Ozgocer *et al*., 2022). Indeed, clinical outcomes showed that males experience both a higher severity and fatality for COVID-19 infection than females ( Mukherjee and Pahan, 2021; Peckham *et al*., 2020; Qu *et al*., 2020).

During the period of follow up, none of the studied participants had documented reinfection with COVID-19, which may be explained by protection provided by neutralizing antibodies. Although the onset of detection and duration of persistence of neutralizing IgM and IgG antibodies differ among studies, they were detectable in sera of most COVID-19 survivors ( Alzaabi *et al*., 2021; Hou *et al*., 2020; Iyer *et al*., 2020; Lee *et al*., 2010; Li *et al*., 2003; Ogega *et al*., 2021; Petersen *et al*., 2021; Qu *et al*., 2020; Sariol and Perlman, 2020).

What amount of the neutralising antibody response could provide protection against infection or re-infection to properly time the vaccination strategy to sustain antibody-mediated protection against SARS-CoV-2 is an important question that must be answered by future studies. Although COVID-19 convalescents or vaccination recipients experienced re-infection, symptomatic re-infections and severe illnesses happened less frequently than in initial infections ( Butt *et al*., 2021; Hacisuleyman *et al*., 2021; Leidi *et al*., 2022) and this is the rationale for studying antibody response against SARS-CoV-2 and implementation of vaccination strategies.

Our study was limited by a small number of participants, and we cannot detect a cutoff value for protecting antibody level. Further studies on larger numbers of participants are required to detect the level of the neutralizing antibody needed to protect against re-infection to correctly schedule the vaccination plan.

## Conclusions

We concluded that the titer of neutralizing IgM and IgG antibodies against COVID-19 virus waned over time until they became negative. In our study, we found a significant reduction in IgM and IgG titers over a period of six months follow up. Regarding younger age, male sex and health care workers, statistically significant persistence of IgM titer was noticed at six months follow up. Also, statistically significant persistent of IgG immunity was found in males and diabetic participants by the end of the six months follow up.

## Consent

Written informed consent for publication of the participants’ details was obtained from the participants.

## Data Availability

Harvard Dataverse: Underlying data for ‘Immunological changes in a cohort of COVID-19 survivors: Mansoura University experience’, https://www.doi.org/10.7910/DVN/O3QGSS ( Khashaba, 2023). This project contains the following underlying data:
- Post Covid project.sav - Post Covid project.xlsx Harvard Dataverse: Extended data for ‘Immunological changes in a cohort of COVID-19 survivors: Mansoura University experience’, https://www.doi.org/10.7910/DVN/O3QGSS ( Khashaba, 2023). This project contains the following extended data:
- COVID 19 sheet final (MUHs)-1.pdf Data are available under the terms of the Creative Commons Zero “No rights reserved” data waiver (CC0 1.0 Public domain dedication).
