# Supplementary material for: Immunological changes in a cohort of COVID-19 survivors: Mansoura University experience
Source: F1000Res. 2023 Aug 29;12:793. Originally published 2023 Jul 6. [Version 2] doi: 10.12688/f1000research.134565.2 (PMC10521065; doi:10.12688/f1000research.134565.2)
Supplement: Peer review history

# Reviewer report: Reviewer response for version 2

**DOI:** 10.5256/f1000research.154910.r201267

**Published:** 2023-09-07

**Authors:** Ritthideach Yorsaeng

**Affiliations:** 1 Center of Excellence in Clinical Virology, Department of Pediatrics, Faculty of Medicine, Chulalongkorn University, Bangkok, Thailand

Thank you for revising the manuscript. The concerns have been addressed.

Is the work clearly and accurately presented and does it cite the current literature?

Yes

If applicable, is the statistical analysis and its interpretation appropriate?

Yes

Are all the source data underlying the results available to ensure full reproducibility?

Yes

Is the study design appropriate and is the work technically sound?

Yes

Are the conclusions drawn adequately supported by the results?

Yes

Are sufficient details of methods and analysis provided to allow replication by others?

Yes

Reviewer Expertise:

Clinical Trials, Vaccine Trials, Immunology, Infectious Diseases

I confirm that I have read this submission and believe that I have an appropriate level of expertise to confirm that it is of an acceptable scientific standard.

# Reviewer report: Reviewer response for version 2

**DOI:** 10.5256/f1000research.154910.r201266

**Published:** 2023-09-05

**Authors:** Zhiwei Chen

**Affiliations:** 1 AIDS Institute and Department of Microbiology, State Key Laboratory of Emerging Infectious Disease, Li Ka Shing Faculty of Medicine, The University of Hong Kong, Hong Kong, Hong Kong

The authors have answered the questions well. No further comments.

Is the work clearly and accurately presented and does it cite the current literature?

Partly

If applicable, is the statistical analysis and its interpretation appropriate?

Not applicable

Are all the source data underlying the results available to ensure full reproducibility?

Yes

Is the study design appropriate and is the work technically sound?

Partly

Are the conclusions drawn adequately supported by the results?

Yes

Are sufficient details of methods and analysis provided to allow replication by others?

Partly

Reviewer Expertise:

Immunity

I confirm that I have read this submission and believe that I have an appropriate level of expertise to confirm that it is of an acceptable scientific standard.

# Reviewer report: Reviewer response for version 1

**DOI:** 10.5256/f1000research.147629.r191284

**Published:** 2023-08-07

**Authors:** Ritthideach Yorsaeng

**Affiliations:** 1 Center of Excellence in Clinical Virology, Department of Pediatrics, Faculty of Medicine, Chulalongkorn University, Bangkok, Thailand

**Major concerns.**

1. Have all study participants not received any COVID-19 vaccination before the study termination?
  Vaccination during the study followed-up may alter the immunologic outcome and distort the results.
2. Have all study participants got re-infection before the study termination?
  Infection during the study followed-up may alter the immunologic outcome and distort the results.

**Minor concerns.**

1. "The assay is a chemiluminescent microparticle immunoassay for qualitative detection of IgG and IgM in human serum or plasma against the SARS-CoV-2 nucleoprotein."
  Are you sure that the IgM assessment in this study targeted anti-nucleocapsid?
2. There was a lacks of information about the immunity trend (immunity waning).

**Comments.**

1. You can add a further keyword, such as; anti-nucleocapsid.
2. "Using an index S/C threshold of 1·4 (for IgG) and 1 (for IgM)."
  Suggest revising a decimal separator to " .".
3. Suggest using one style of English. I found this manuscript contained both UK and US styles.
  For example, neutralising and neutralizing.
4. The age group can be stratified (adolescent, young adult, middle-aged and elderly) to see more immunity waning trend in all Tables and Figures. Because a young age has higher immunity than an elder. If it works.

Is the work clearly and accurately presented and does it cite the current literature?

Yes

If applicable, is the statistical analysis and its interpretation appropriate?

Yes

Are all the source data underlying the results available to ensure full reproducibility?

Yes

Is the study design appropriate and is the work technically sound?

Yes

Are the conclusions drawn adequately supported by the results?

Yes

Are sufficient details of methods and analysis provided to allow replication by others?

Yes

Reviewer Expertise:

Clinical Trials, Vaccine Trials, Immunology, Infectious Diseases

I confirm that I have read this submission and believe that I have an appropriate level of expertise to confirm that it is of an acceptable scientific standard, however I have significant reservations, as outlined above.

# Response

**Published:** 2023-08-20

**Authors:** Eman Khashaba

**Affiliations:** Mansoura University, Egypt

**Major concern**s.

**Have all study participants not received any COVID-19 vaccination before the study termination? Vaccination during the study followed-up may alter the immunologic outcome and distort the results.**

**Reply:** None of the participants received COVID-19 vaccination before the study termination as our study was conducted post COVID-19 infection and the participants were not convinced to receive any vaccination before at least one-year post infection. This was proved by history taking during follow up.

**Have all study participants got re-infection before the study termination? Infection during the study followed-up may alter the immunologic outcome and distort the results.**

**Reply:** Re-infection was excluded based on review of clinical symptoms during follow up visits. This was mentioned in discussion.

**Minor concerns** **.**

**"The assay is a chemiluminescent microparticle immunoassay for qualitative detection of IgG and IgM in human serum or plasma against the SARS-CoV-2 nucleoprotein."**

**Reply:** The amount of IgG and IgM antibodies to SARS-CoV-2 in each sample is determined by its chemiluminescent relative light unit (RLU) to the calibrator RLU using an index S/C threshold of 1.4 for IgG and 1.0 for IgM as stated on page 3

**Are you sure that the IgM assessment in this study targeted anti-nucleocapsid?**

**Reply:** Yes, the chemiluminescent microparticle assay detects the IgM targeted against the SARS-CoV-2 nucleocapsid protein

**There was a lacks of information about the immunity trend (immunity waning).**

 Reply: The authors illustrated in figures the immunity waning according to measured levels of  I g M and IgG at 1,3,6  months ( **Table1, Figure 2 &3**) in results section.

**Comments.**

**You can add a further keyword, such as; anti-nucleocapsid.**

 Ok. The keyword is added

**"Using an index S/C threshold of 1·4 (for IgG) and 1 (for IgM)."**

**Suggest revising a decimal separator to " .".**

 Ok. Done

**Suggest using one style of English. I found this manuscript contained both UK and US styles.**

 For example, neutralising and neutralizing.

 Ok done UK style

**The age group can be stratified (adolescent, young adult, middle-aged and elderly) to see more immunity waning trend in all Tables and Figures. Because a young age has higher immunity than an elder. If it works.**

 Reply: our study results revealed that there was statistically significant difference according to mean age between positive and negative groups (Table 2). Based on these results ,we did logistic regression analysis which revealed that increased age (continuous variable) is associated with decreased chance of  positive IgM ONLY (Table 4) . These results agree with the reviewer' s comments that young age is associated with better immunity (necessary changes will be made in discussion).

# Reviewer report: Reviewer response for version 1

**DOI:** 10.5256/f1000research.147629.r187529

**Published:** 2023-07-26

**Authors:** Zhiwei Chen

**Affiliations:** 1 AIDS Institute and Department of Microbiology, State Key Laboratory of Emerging Infectious Disease, Li Ka Shing Faculty of Medicine, The University of Hong Kong, Hong Kong, Hong Kong

In this study, Tamer Elhadidy et al. reported the anti-NP IgM and IgG titer in individuals at 1-, 3- and 6-months post SARS-CoV-2 infection and further explain the correlation between antibody and disease severity, gender and diabetes. There are several major issues that need to be addressed.

  

1. The authors measured IgG and IgM titer against SARS-CoV-2 NP, not the spike. However, neither anti-NP IgM nor IgG have neutralizing activity. Although the results provide some information on humoral responses, the value for vaccine-mediated protection is limited. The authors should discuss this limitation of the study.
2. In method of the Chemiluminescent immunoassay, the authors mention that they used human serum or plasma for IgG and IgM measurements. It should be noted that plasma was isolated from the vacutainer contained EDTA or heparin. There might be a difference in dilution factor depending on the isolation methods used. It is not clear if such dilution factor would affect the results with 2 times difference between IgG_1m and IgG_6m (Figure 3). The authors should clarify this.
3. There were no standard assays used in this study for comparison with other studies. WHO already established the International Standard for anti-SARS-CoV-2 immunoglobulin ( https://cdn.who.int/media/docs/default-source/biologicals/bs-documents-(ecbs)/2022-documents/new-2022-document-susan/bs-2022.2427_mattiuzzo-g._sars-cov-2_ab_2ndisandrpfor-voc_final.pdf?sfvrsn=90585abb_1&download=true). The authors may test some samples using a standard assay to increase the value of this study.
4. What’s the detection limit of the IgM and IgG assay or how to define the positive and negative of the test? In Table 2 and Table 3, the authors separated the positive test and negative test for comparison. Can the authors explain?
5. Human serum or plasma samples isolated from individuals without infection history should be included as cutoff values.  Since NP is quite conserve between common cold coronavirus and SARS-CoV-2, the issue of antibody cross reactivity should be addressed.

Is the work clearly and accurately presented and does it cite the current literature?

Partly

If applicable, is the statistical analysis and its interpretation appropriate?

Not applicable

Are all the source data underlying the results available to ensure full reproducibility?

Yes

Is the study design appropriate and is the work technically sound?

Partly

Are the conclusions drawn adequately supported by the results?

Yes

Are sufficient details of methods and analysis provided to allow replication by others?

Partly

Reviewer Expertise:

Immunity

I confirm that I have read this submission and believe that I have an appropriate level of expertise to confirm that it is of an acceptable scientific standard, however I have significant reservations, as outlined above.

# Response

**Published:** 2023-08-20

**Authors:** Eman Khashaba

**Affiliations:** Mansoura University, Egypt

**Comment 1:** The authors measured IgG and IgM titer against SARS-CoV-2 NP, not the spike. However, neither anti-NP IgM nor IgG have neutralizing activity. Although the results provide some information on humoral responses, the value for vaccine-mediated protection is limited. The authors should discuss this limitation of the study.

**Reply: **Vaccine mediated immunity was not studied in current research as all study subjects were post-Covid and none of them received vaccination. This note will be added to limitations of study after discussion.

**Comment 2:** in method of chemiluminescent immunoassay, the authors mentioned that they used human serum or plasma IgG and IgM. It should be noted that plasma was isolated from vacutainer contained EDTA or heparin there might be difference in dilution factor There might be a difference in dilution factor depending on the isolation methods used. It is not clear if such dilution factor would affect the results with 2 times difference between IgG_1m and IgG_6m (Figure 3). The authors should clarify this.…….

**Reply:** All samples included in the study were serum samples, the chemiluminescent microparticle immunoassay can detect IgG and IgM in human serum or plasma but only serum samples were included in this study as stated on page 3 so there was no need for a dilution factor …….

**Comment 3: **There were no standard assays used in this study for comparison with other studies. WHO already established the international standard for anti-SARS-CoV-2 immunoglobulin….. ( https://cdn.who.int/media/docs/default-source/biologicals/bs-documents-

 (ecbs)/2022-documents/new-2022-document-susan/bs-2022.2427_mattiuzzo-g._sars-cov-2_ab_2ndisandrpfor-voc_final.pdf?sfvrsn=90585abb_1&download=true). The authors may test some samples using a standard assay to increase the value of this study.

**Reply:** We have used FDA approved standardized commercial kits from Abbott laboratories with provided calibrators used in calculation of antibody levels

**Comment 4:** What is the detection limit of IgM and IgG assay or how to define the positive or negative …… In Table 2 and Table 3, the authors separated the positive test and negative test for comparison. Can the authors explain?

**Reply: **The amount of IgG and IgM antibodies to SARS-CoV-2 in each sample is determined by its chemiluminescent relative light unit (RLU) to the calibrator RLU using an index S/C threshold of 1.4 for IgG and 1.0 for IgM as stated on page 3

**Comment 5:** Human serum or plasma samples isolated from individuals without infection history should be included as cutoff values.  Since NP is quite conserve between common cold coronavirus and SARS-CoV-2, the issue of antibody cross reactivity should be addressed.

**Reply: **We used FDA approved standardized commercial kits from Abbott laboratories with provided calibrators used in calculation of antibody levels. Cross reactivity could not be addressed in this study. This comment was added to limitations of study
